# *Acidipropionibacterium acidipropionici*, a propionate-producing bacterium, contributes to GPR41 signaling and metabolic regulation in high-fat diet-induced obesity in mice

**DOI:** 10.3389/fnut.2025.1542196

**Published:** 2025-04-03

**Authors:** Junki Miyamoto, Yuna Ando, Mayu Yamano, Akari Nishida, Kota Murakami, Ikuo Kimura

**Affiliations:** ^1^Department of Applied Biological Science, Graduate School of Agriculture, Tokyo University of Agriculture and Technology, Fuchu-shi, Tokyo, Japan; ^2^Laboratory of Molecular Neurobiology, Graduate School of Biostudies, Kyoto University, Kyoto, Japan; ^3^Department of Molecular Endocrinology, Graduate School of Pharmaceutical Sciences, Kyoto University, Kyoto, Japan

**Keywords:** *Acidipropionibacterium acidipropionici*, short-chain fatty acids, propionate, obesity, GPR41

## Abstract

Obesity is a major healthcare problem worldwide and is induced by excess energy intake, resulting in gut microbial composition and microbial diversity changes. Through fermentation of dietary fibers, short-chain fatty acids (SCFAs) act as host energy sources and signaling molecules via G protein-coupled receptors such as GPR41. *Acidipropionibacterium acidipropionici* is widely used in many applications; however, *in vivo* studies on the beneficial effect of *A. acidipropionici* via propionate production and host energy homeostasis are unclear. Therefore, this study aimed to investigate the beneficial metabolic effects of *A. acidipropionici* by focusing on GPR41 signaling in a high-fat diet (HFD)-induced obesity mouse model. Here, we demonstrated that *A. acidipropionici* OB7439 improved host metabolism in HFD-induced obesity in mice. The intake of *A. acidipropionici* OB7439 improved metabolism in HFD-induced obese mice by increasing propionate production, regulating glucose tolerance, and inhibiting hepatic inflammation via GPR41 signaling. Our findings shed light on the potential of using *A. acidipropionici* OB7439 as an SCFA producer for the prevention and treatment of metabolic disorders. Based on these results, we suggest that *A. acidipropionici* may be a potential therapeutic bacterium that inhibits obesity and modulates the gut microbial community.

## 1 Introduction

*Acidipropionibacterium acidipropionici* is an anaerobic Gram-positive bacterium that is widely used for many applications, such as propionic acid production, owing to its ability to utilize glucose and fructose (1, 2). During fermentation, *A. acidipropionici* produces propionic acid via two main fermentation pathways; pyruvate (the Wood–Werkman cycle) and lactate (the acrylate pathway) (3, 4). Propionic acid is a product of great interest in the food, feed, pharmaceutical, cosmetic, and plastic industries (3, 5). Various bacterial metabolites, particularly short-chain fatty acids (SCFAs), exert beneficial effects on human health (6, 7). SCFAs (primarily acetate, propionate, and n-butyrate) are bacterial metabolites produced via complex carbohydrate fermentation. Gut microbial SCFAs act as host energy sources and signal molecules via host receptors, such as the G protein-coupled receptors (GPCRs) GPR41 and GPR43, which improve host homeostasis by affecting the endocrine systems. GPR41 affects host metabolic function by increasing sympathetic activity and promoting gut hormone secretion, whereas GPR43 suppresses fat accumulation and promotes gut hormone secretion (8).

Diet is the most important energy resource for daily activities and is crucial in influencing metabolism. An imbalance between energy consumption and expenditure is the fundamental cause of obesity (9). Obesity is a serious threat to global health as it is associated with many metabolic diseases and is caused by interactions between genetic, environmental, and psychosocial factors (10). In particular, excess food intake, the composition of high-fat and high-carbohydrate diets, insufficient exercise, and genetic factors are considered risk factors for obesity (11). However, several other studies have suggested that the gut microbiota plays a crucial role in regulating energy homeostasis. Moreover, changes in the gut microbiota can lead to dysbiosis, which occurs during obesity. Weight gain and metabolic disorders were observed when the gut microbiota of obese mice were inoculated into normal or germ-free mice (12). Concordantly, dysbiosis has been implicated in several systemic disorders, including the metabolic syndrome. Specific gut microbiota is associated with obesity, and the ratio of representative dominant strains of Firmicutes and Bacteroidota is one of the main factors. Changes in the gut microbial community due to obesity are related to the ability of the microbes to harvest dietary energy and alter fatty acid metabolism in the adipose tissue and liver (13, 14). Changes in dominant strains alter energy harvesting and subsequently cause metabolic problems. Therefore, modulation of the gut microbiota composition by probiotics represents an attractive and potential therapy for metabolic disorders (15).

Probiotics is defined “live microorganisms which when intake in adequate amounts can benefit on host health” (16). Probiotic intervention for dysbiosis can change the composition of the gut microbiota and thus reduce obesity-related symptoms, such as fat and cholesterol levels as well as weight gain (17, 18). Previous studies have found that probiotics can influence obesity by affecting insulin sensitivity and inflammatory pathways by modulating gut microbiota compositions (19). Additionally, probiotic therapy is widely used to treat metabolic diseases, such as obesity (20, 21). Current research on the use of probiotics for the treatment of obesity-related disorders has been conducted primarily using preclinical models. As a next-generation probiotics, *Akkermansia muciniphila* is focused on its role and growing its importance on obesity. *A. muciniphila* is isolated from human feces, a strict anaerobe Gram-negative bacterium, and utilize mucin as its sole carbon, nitrogen, and energy source (22). In humans, studies have provided evidence for a negative correlation between Akkermansia muciniphila abundance and overweight, obesity, type 2 diabetes, or hypertension (23). Previous study provide a promising signal for the development of future clinical intrerventions and the impact of oral supplementation with *A. muciniphila* in overweight or obese insulin-resistant individuals. Additionally, *Lactobacillus plantarum* is present in many fermented foods. *Lactobacillus plantarum* treatment improved NAFLD and reduced obesity in animal models (24, 25). Lactobacillus paracasei is isolated from the intestinal tracts of infants and improves fatty liver and lipid metabolism (26). However, the effects of *A. acidipropionici* on propionate production and the regulation of energy homeostasis remain unclear. Therefore, in this study, we investigated the beneficial metabolic effects of *A. acidipropionici* via GPR41 signaling in a high-fat diet (HFD)-induced obesity mouse model.

## 2 Materials and methods

### 2.1 Bacterial culture

*Acidipropionibacterium acidipropionici* OB7439 was kindly provided by the Food Science Institute Foundation (Ryoushoku Kenkyukai). *A. acidipropionici* JCM6427 was obtained from the Japan Collection of Microorganisms (JCM; Saitama, Japan). Each strain was cultured in GAM broth (Nissui Pharmaceuticals, Tokyo, Japan) in screw-capped tubes under anaerobic conditions using AnaeroPack (Mitsubishi Gas Chemical, Tokyo, Japan) at 37°C for 2 days. After cultivation, cells and supernatants were separated by centrifugation (4°C, 1,500 × g for 10 min), and the supernatants were collected to measure SCFA levels.

### 2.2 Animal study

Conventional C57BL/6J (SLC Inc., Shizuoka, Japan) and *Gpr41^–/–^* mice were housed under a 12-h light–dark cycle and provided with normal chow (CE-2; CLEA, Tokyo, Japan). *Gpr41^–/–^* mice were generated as previously described and maintained on a C57BL/6J genetic background (27). Germ free (GF)-ICR mice were housed in vinyl isolators under a 12-h light–dark cycle and given normal chow (CL-2, 50 kGy irradiated; CLEA) and an autoclaved water *ad libitum*. GF-ICR mice were routinely analyzed for sterility by culturing under aerobic and anaerobic conditions and qPCR analysis (16S rRNA gene) (28). All mice were sacrificed under deep isoflurane-induced anesthesia. All efforts were made to minimize suffering.

All experimental procedures involving mice were performed following protocols approved by the Committee on the Ethics of Animal Experiments of the Tokyo University of Agriculture and Technology (permit number: R5–48). The study was conducted in accordance with the ARRIVE guidelines and all methods were performed following the American Veterinary Medical Association (AVMA) Guidelines for the Euthanasia of Animals (29). The principles established in the institutional guidelines were faithfully followed during the duration of this study.

To evaluate the glucose absorption and metabolism by acute oral administration, 16 h–fasted GF-ICR (male, 7-week-old) and C57BL/6J (male, 7-week-old) mice were orally administered (p.o.) glucose (2 g/kg of body weight) including OB7439 or JCM6427 (1 × 10^10^ cfu/mouse) (30). For the glucose tolerance test (GTT), blood glucose levels were monitored before and 15, 30, 60, and 120 min after injection.

For a high-fat diet (HFD) loading, 4-week-old C57BL/6J and *Gpr41^–/–^* male mice were fed a D12492 diet (control; 60% kcal fat; Research Diets) or OB7439-supplemented D12492 diet [OB7439; 1 × 10^7^ cfu/g, (31, 32)] for 12 weeks (33–37). The diets were replaced with fresh diets to keep the bacterial alive once every 2 days. To evaluate the effect on glucose tolerance by intraperitoneally GTT, 16 h–fasted obese mice were intraperitoneally injected (i.p.) with glucose (2 g/kg of body weight) (38–40). For the insulin tolerance test (ITT), 3 h–fasted obese mice were intraperitoneally injected (i.p.) with human insulin (0.75 mU/kg of body weight; Sigma-Aldrich, St. Louis, MO, United States). Blood glucose levels were monitored before and 15, 30, 60, 90, and 120 min after injection.

### 2.3 Short-chain fatty acids measurement

SCFA content in the culture supernatants by *A. acidipropionici* OB7439 or JCM6427 and murine plasma, cecum, and feces was determined using a modification of a protocol described previously (41). Briefly, culture supernatant, plasma, cecum, and feces were immediately treated with 5-sulfosalicylic acid, followed by 1 min of vortex-mixing. The samples were centrifuged at 15,000 × g for 15 min, and the supernatant was collected. The supernatant was added to 2-ethylbutyric acid (as an internal control), hydrochloric acid, and diethyl ether, followed by 1 min of vortex-mixing. The samples were centrifuged at 3,000 × g for 5 min, and the SCFA-containing ether layers were collected and pooled for gas chromatography-mass spectrometry (GC-MS) analysis using a GCMS-QP2010 Ultra (Shimadzu, Kyoto, Japan). The VF-WAXms column (30 m × 0.25 mm internal diameter × 1 μm; Agilent technologies) was used for chromatographic separation. Helium (0.92 mL/min) was used as the carrier gas. The mass spectrometer was set to scan mode from m/z 40–130 and in selected ion monitoring mode at m/z of 60 (retention time; 9.6) for acetate, m/z of 74 (retention time; 10.7) for propionate, m/z of 60 (retention time; 12.5) for n-butyrate, and m/z of 88 (retention time; 13.6) for 2-ethylbutyric acid. Calibration curves for the SCFAs were plotted and their concentrations in each sample were evaluated over a specified concentration range.

### 2.4 Biochemical analyses

Blood glucose levels were measured by using One Touch Ultra (LifeScan, Milpitas, CA). The levels of plasma triglyceride (TG, LabAssay™ Triglyceride; FUJIFILM Wako Pure Chemical Co., Ltd., Osaka, Japan), non-esterified fatty acid (NEFA, LabAssay™ NEFA; FUJIFILM Wako Pure Chemical Co., Ltd.), total cholesterol (t-cho, LabAssay™ Cholesterol; FUJIFILM Wako Pure Chemical Co., Ltd.), active glucagon-like peptide-1 (GLP-1, GLP-1 [Active] ELISA; Merck Millipore, Billerica, MA), total glucose-dependent insulinotropic polypeptide (GIP, Rat/Mouse GIP (total) ELISA, Merck Millipore), peptide YY (Mouse/Rat PYY ELISA Kit; FUJIFILM Wako Pure Chemical Co., Ltd.), and insulin [Mouse Insulin enzyme-linked immunosorbent assay (ELISA); Shibayagi, Gunma, Japan] were measured according to the manufacturer’s instructions. For active GLP-1 and total GIP measurements, plasma samples were treated with a dipeptidyl peptidase IV inhibitor (Merck Millipore) to prevent the degradation of active GLP-1 and GIP.

### 2.5 RNA extraction and quantitative reverse transcription-polymerase chain reaction

Total RNA was isolated from the liver using the RNAiso Plus reagent (TaKaRa, Shiga, Japan). cDNA was transcribed using RNA as templates and Moloney murine leukemia virus reverse transcriptase (Invitrogen, Carlsbad, CA, United States). The RT-qPCR analysis was performed using a StepOnePlus real-time PCR system (Applied Biosystems, Foster City, CA, United States) with TB Green Premix Ex Taq II (TaKaRa). The reaction was performed at 95°C for 30 s, followed by 40 cycles of 95°C for 5 s, 58°C for 30 s, and 72°C for 1 min. The dissociation stage was analyzed at 95 °C for 15 s, followed by one cycle of 60°C for 1 min and 95°C for 15 s. The sequences of the primers used were as follows: *Tnfa*, 5′-TCGTAGCAAACCACCAAGTG-3′ (forward) and 5′-CTTTGAGATCCATGCCGTTG-3′ (reverse); *F4/80*, 5′-GGAGGATGGGAGATGGACAC-3′ (forward) and 5′-ACAGC ACGACACAGCAGGAA-3′ (reverse); *Col1a*, 5′- CCTCAGGGT ATTGCTGGACAAC-3′ (forward) and 5′- ACCACTTGATCCA GAAGGACCTT-3′ (reverse); *Fas*, 5′-GCTGCGGAAACTTCAG GAAAT-3′ (forward) and 5′-AGAGACGTGTCACTCCTGGACTT-3′ (reverse); *Chrebp*, 5′-CTGGGGACCTAAACAGGAGC-3′ (for ward) and 5′-GAAGCCACCCTATAGCTCCC-3′ (reverse); *Ppara*, 5′-CCTGAACATCGAGTGTCGAA-3′ (forward) and 5′-GGCCT TGACCTTGTTCATGT-3′ (reverse); *Tbet*, 5′-ACCAGAACGCA GAGATCACTCA-3′ (forward) and 5′-CAAAGTTCTCCCGGA ATCCTT-3′ (reverse); *Gata3*, 5′-GTCATCCCTGAGCCACATCT-3′ (forward) and 5′-AGGGCTCTGCCTCTCTAACC-3′ (rever se); *Rorc*, 5′-TGCAAGACTCATCGACAAGGC-3′ (forward) and 5′-AGCTTTTCCACATGTTGGCTG-3′ (reverse); *Foxp3*, 5′-CT GGGTTTAAGATCCCAGCA-3′ (forward) and 5′-GAGAGGCC TAGAGCCCTGAT-3′ (reverse); and *18S*, 5′-ACGCTGAGC CAGTCAGTGTA-3′ (forward) and 5′-CTTAGAGGGACA AGTGGCG-3′ (reverse).

### 2.6 Histology

The liver was embedded in OCT compound (Sakura Finetek, Osaka, Japan) and sectioned into 7 μm thick slices. Hematoxylin and eosin staining was performed using standard techniques, with minor modifications (42, 43).

### 2.7 Gut microbial composition

Cecal and fecal DNA was extracted from frozen samples using the FastDNA SPIN kit for feces (MP Biomedicals, Santa Ana, CA, United States) as described previously (44). Partial 16S rRNA gene sequences were amplified by targeting the hypervariable regions v4 using the primers 515F, 5′-TCGTCGGCAGCGTCAGATGTGTATAAGAGACAGGTGYCAG CMGCCGCGGTAA-3′ and 806R, 5′-GTCTCGTGGGCTCGG AGATGTGTATAAGAGACAGGGACTACHVGGGTWTCTAAT-3′. Amplicons generated from each sample were subsequently purified using AMPure XP (Beckman Coulter, Brea, CA, United States) and sequenced using a MiSeq sequencer (Illumina, San Diego, CA, United States) and MiSeq Reagent kit (version 3.0; 600 cycles). The 16S rRNA sequence data were processed using the quantitative insights into microbial ecology (QIIME 2. 2022.2.0)^1^ pipeline and diversity analyses were performed using the QIIME script core_diversity_analyses.py. The statistical significance of sample groupings was assessed using permutational multivariate analysis of variance (ANOVA; QIIME script compare_categories.py). Firmicutes and Bacteroidota, identified at the phylum level, were used to determine the ratio of Firmicutes and Bacteroidota. Moreover, qPCR was performed using SYBR Premix Ex Taq II (Takara Bio) and the StepOnePlus real-time PCR system (Applied Biosystems). The primer sequences were as follows: *A. acidipropionici*, 5′-GAGGGGATGAGCTGTGGATA-3′ (forward) and 5′-CATTCGGAGTTTGGCTGATT-3′ (reverse).

### 2.8 Statistical analyses

All values are presented as the mean ± standard error. Data were analyzed using the commercially available statistical software Prism version 9.4.1 (GraphPad Software, La Jolla, CA).^2^ Data normality was checked using the Shapiro–Wilk test. For normal data, an equal-variance test was performed using the F test (for comparing two groups) or Bartlett′s test (for comparing more than three groups). The Mann–Whitney test (nonparametric data) was used to compare two groups. One-way ANOVA (for parametric data) or the Kruskal–Wallis test (for nonparametric data) was performed when comparing more than three groups, followed by *post hoc* multiple comparison tests (either Dunnett’s test or Dunn’s test). The *P*-values from these multiple comparison tests were corrected and reported as adjusted *P*-values. Statistical significance was set at *P* < 0.05.

## 3 Results

### 3.1 *Acidipropionibacterium acidipropionici* OB7439 produced SCFAs *in vitro* and *in vivo*

**FIGURE 1:**
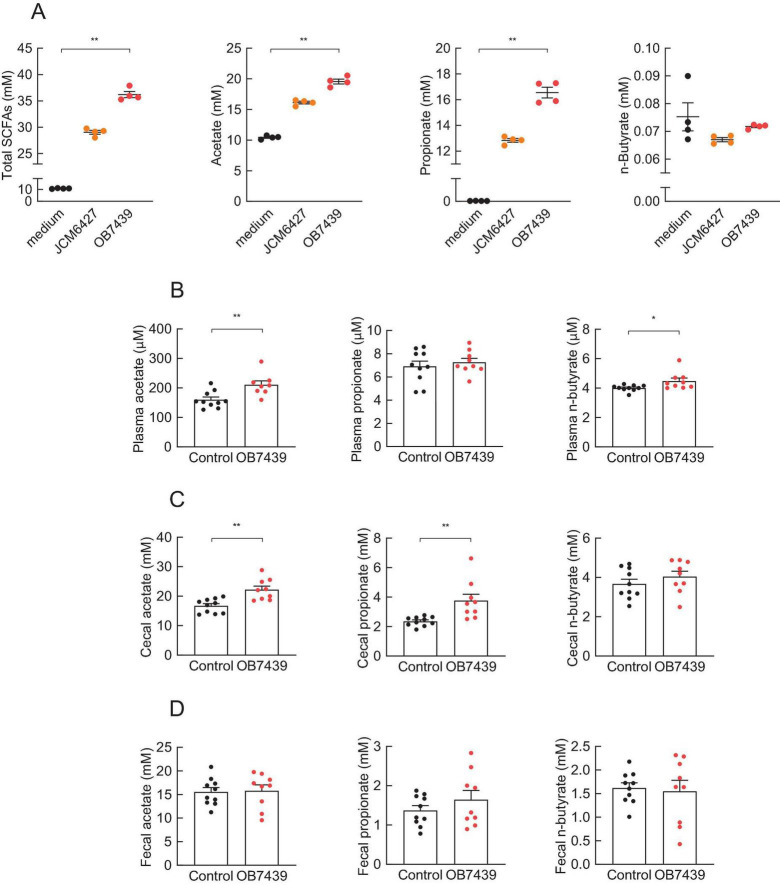
Short-chain fatty acid (SCFA) production in culture medium and mice. **(A)** SCFA levels in the culture medium (*n* = 4 per group); OB7439, *Acidipropionibacterium acidipropionici* OB7439; JCM6427, *Acidipropionibacterium acidipropionici* JCM6427; medium, GAM medium without OB7439 or JCM6427. SCFA levels in the **(B)** plasma, **(C)** cecum, and **(D)** feces of high fat diet (HFD)-fed mice (*n* = 10 per group); OB7439, HFD diet with OB7439 supplementation (1 × 10^7^ cfu/g); Control, HFD diet without OB7439 supplementation. All SCFA levels were measured using GC-MS. **P < 0.01, *P < 0.05 in Dunn’s *post-hoc* test for **(A)** and Mann–Whitney test for **(B–D)**. Results are presented as the mean ± standard error of the mean (SEM).

To investigate whether *Acidipropionibacterium acidipropionici* OB7439 (OB7439) produces SCFAs *in vitro* and *in vivo*, we measured SCFA production following OB7439 cultivation and intake. After cultivation for 2 days, the SCFA production (total, acetate, and propionate levels) was remarkably increased in the OB7439-cultured supernatants compared with that in the medium-only (Figure 1A). Moreover, the SCFA production tended to increase in the OB7439-cultured supernatants compared with that in the JCM6427-cultured supernatants (Figure 1A). Additionally, plasma, cecal, and fecal levels of SCFAs in the OB7439-supplemented HFD-fed mice were significantly higher than those in HFD-fed control mice (Figures 1B–D). In particular, the levels of cecal acetate and propionate were significantly increased in OB7439-supplemented HFD-fed mice and were significantly higher than those in HFD-fed control mice (Figure 1C). Thus, OB7439 intake markedly increased SCFA production in the cecum (Figure 1C).

### 3.2 *Acidipropionibacterium acidipropionici* OB7439 regulated glucose tolerance

**FIGURE 2:**
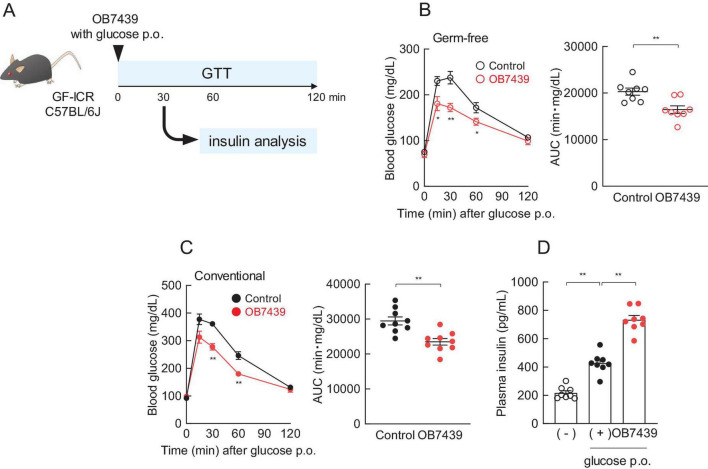
Regulation of glucose tolerance by *Acidipropionibacterium acidipropionici* OB7439. **(A)** Experimental scheme. **(B)** Blood glucose levels in germ free (GF)-ICR mice (*n* = 8 per group) and **(C)** conventional C57BL/6J mice (*n* = 8 per group) according to oral glucose tolerance test (GTT) and area under the curve (AUC) analyses of GTT performed after 16-h fasting. **P < 0.01, *P < 0.05, compared to the control in Mann–Whitney test. **(D)** Plasma insulin levels were measured 30 min after oral glucose administration (p.o) in C57BL/6J mice (*n* = 8 per group). (—) Indicates only fasting, and (+) indicates only glucose p.o. **P < 0.01, *P < 0.05 (Dunn’s *post-hoc* test). Results are presented as the mean ± standard error of the mean (SEM).

Considering that an increase in the SCFA production was observed in the OB7439 supernatants and OB7439-supplemented HFD-fed mice, we investigated the beneficial effects of glucose tolerance. To evaluate the effect of SCFAs derived from OB7439, we performed a glucose tolerance test (GTT) in germ-free (GF) mice (Figure 2A). After 16-h fasting, GF mice were orally administered glucose with or without OB7439. We found that OB7439 administration significantly suppressed increases in blood glucose levels compared with that observed in control GF mice (Figure 2B). However, the effect of heat-killed OB7439 administration was similar between control mice (data not shown). Additionally, OB7439 administration improved blood glucose levels in conventional C57BL/6J mice compared with those in control mice (Figure 2C). Moreover, following glucose administration at 30 min, the levels of glucose-induced insulin secretion in OB7439-administered mice were higher than those in control mice (Figure 2D). However, the effect of JCM6427 administration was similar between GF and conventional control mice (Supplementary Figure 1). In correlation with the SCFA production shown in Figure 1A, acute administration of OB7439 significantly promoted glucose-induced insulin secretion and improved glucose intolerance than acute administration of JCM6427.

### 3.3 *Acidipropionibacterium acidipropionici* OB7439 intake improved HFD-induced obesity

**FIGURE 3:**
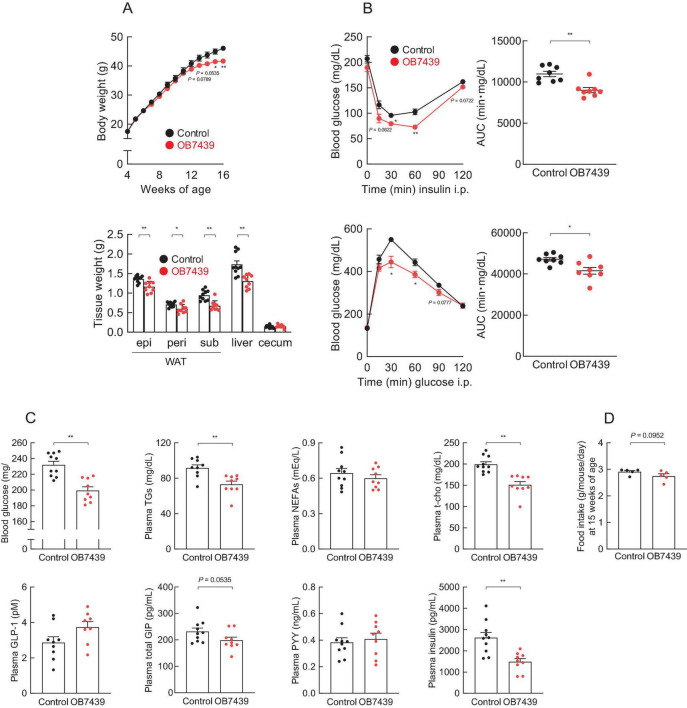
*Acidipropionibacterium acidipropionici* OB7439 improves host metabolic condition. **(A–C)** C57BL/6J male mice were fed a high-fat diet (HFD, Control) or *Acidipropionibacterium acidipropionici* OB7439 (1 × 10^7^ cfu/g)-supplemented HFD (OB7439) for 12 weeks. **(A)** Body and tissue weights. epi, epididymal; peri, perirenal; sub, subcutaneous; WAT, white adipose tissue. **(B)** Insulin tolerance test (ITT) after intraperitoneal (i.p.) injection and area under the curve (AUC) analyses of ITT (*upper*, *n* = 8–10 per group) were performed at 13–14 weeks of age. Glucose tolerance test (GTT) after intraperitoneal (i.p.) injection and AUC analyses of GTT (*lower*, *n* = 8–10 per group) were performed at 13–14 weeks of age. **(C)** Blood glucose, plasma triglycerides (TGs), non-esterified fatty acids (NEFAs), total cholesterol (t-cho), glucagon-like peptide-1 (GLP-1), glucose-dependent insulinotropic polypeptide (GIP), peptide YY (PYY), and insulin levels were measured at the end of the experimental period (*n* = 10 per group). **(D)** The daily food intake at 15 weeks of age (*n* = 5 per group). **P < 0.01 and *P < 0.05 (Mann–Whitney U-test). Results are presented as the mean ± standard error of the mean (SEM).

We have previously reported that SCFA (e.g., propionate) intake affects host energy homeostasis in HFD-induced obese mice (33, 45). Next, we investigated the beneficial metabolic effects of OB7439 supplementation in HFD-induced obese mice. We found that the body weight of OB7439-fed mice was significantly lower than that of HFD-fed control mice during growth (Figure 3A, *left*). Additionally, the white adipose tissue and the liver weights were significantly lower in OB7439-fed mice than in HFD-fed control mice at 16 weeks of age (Figure 3A, *right*). Moreover, HFD-induced insulin resistance and impaired glucose tolerance, as determined by the insulin tolerance test (ITT) and GTT, respectively, were significantly attenuated in OB7439-fed mice as compared with those in HFD-fed control mice (Figure 3B). Furthermore, blood glucose, plasma triglycerides (TGs), and total cholesterol (t-cho) levels of OB7439-fed mice were significantly lower than those of HFD-fed control mice (Figure 3C), whereas non-esterified fatty acids (NEFAs), active glucagon-like peptide-1 (GLP-1), and peptide YY (PYY) levels were similar between HFD-fed control and OB7439-fed mice (Figure 3C). Additionally, we found that plasma glucose-dependent insulinotropic polypeptide (GIP) and insulin levels in OB7439-fed mice were slightly lower than those in HFD-fed control mice (Figure 3C). Moreover, the food intake in OB7439-fed mice tended to be lower than that in control mice (Figure 3D). Therefore, OB7439 supplementation suppressed body mass increase and improved metabolic conditions, thereby inducing greater resistance to HFD-induced obesity.

We have previously reported that the intake of SCFA (e.g., propionate) improves hepatic metabolic conditions (33, 34). Hence, we further examined changes in hepatic lipid metabolism. The hepatic TG levels in OB7439-fed mice were significantly lower than those in HFD-fed control mice (Supplementary Figures 2A,B, *left*). However, hepatic t-cho levels were similar between HFD-fed control and OB7439-fed mice (Supplementary Figure 2B, *right*). We also investigated the expression profiles of hepatic genes related to inflammation and energy metabolism. The hepatic mRNA expression levels of the inflammatory marker *Tnf*α, the macrophage marker *F4/80*, and the fibrosis marker *Col1a* were significantly decreased in OB7439-fed mice compared with those in HFD-fed control mice (Supplementary Figure 2C, *upper*). Moreover, *Fas* and *Chrebp* (fatty acid synthesis) mRNA expression levels were markedly decreased, whereas those of *Ppara* (lipid metabolism) tended to increase in OB7439-fed mice compared with those in HFD-fed control mice (Supplementary Figure 2C, *lower*). A commensal propionate-producing bacterium, Propionibacterium genus, mitigated intestinal inflammation via Th17 cell regulation and contributed to immune regulation that may prevent intestinal pathogen infection (46–48). Here, we evaluated the effects of immune modulation in the different propionate-producing bacterium, *Acidipropionibacterium acidipropionici* OB7439; however, the mRNA expression of T cell transcription markers in the liver of HFD-fed control mice was comparable with that in OB7439-fed mice (Supplementary Figure 2D). Thus, OB7439 supplementation suppressed the HFD-induced accumulation of hepatic TGs by changing hepatic lipid metabolism.

### 3.4 *Acidipropionibacterium acidipropionici* OB7439 improved gut microbial composition

**FIGURE 4:**
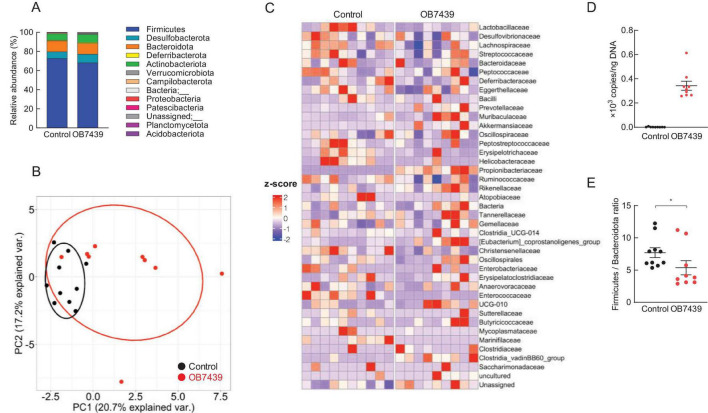
*Acidipropionibacterium acidipropionici* OB7439 changes gut microbial composition in the cecum of obese mice. **(A)** Relative abundance of gut microbiota at the phylum level, **(B)** principal coordinate analysis, **(C)** abundance of gut microbiota at the family level, **(D)** levels of *A. acidipropionici*, and **(E)** Firmicutes to Bacteroidota ratio (*n* = 9–10). Data are presented as means ± standard error of the mean (SEM). Control, mice fed high-fat diet (HFD); OB7439, mice fed HFD supplemented with *Acidipropionibacterium acidipropionici* OB7439 (1 × 10^7^ cfu/g). *P < 0.05 (Mann–Whitney U-test).

We then examined the effects of OB7439 on the gut microbial composition in HFD-induced obese mice. Taxonomic analysis of the gut microbiota in the cecum showed that the bacterial population was similar between HFD-fed control and OB7439-fed mice (Figure 4A). However, hierarchical clustering and heatmap of individual families in the cecum were slightly different between HFD-fed control and OB7439-fed mice (Figures 4B,C). Moreover, the level of *A. acidipropionici* was significantly increased in OB7439-fed mice compared with those in HFD-fed control mice (Figure 4D). In agreement with a previous study (12), we observed increased Firmicutes and decreased Bacteroidota levels in HFD-fed mice; however, OB7439-fed mice had decreased Firmicutes and increased Bacteroidota levels (Figure 4E). In contrast, changes in the gut microbiota in feces following OB7439 intake were less pronounced than in the cecum (Supplementary Figure 3). Moreover, *A. acidipropionici* is belonging to Propionibacteriaceae family and the levels of Propionibacteriaceae family in the cecum and feces were detected in only OB7439-fed mice (Figure 4B; Supplementary Figure 3B). Additionally, we investigated the PICRUSt2 analysis in the cecal and fecal microbiota. Although no drastic changes in the OB7439-specific pathways involved in SCFAs production were observed, some metabolism pathways in OB7439-supplemented HFD-fed mice were changed compared with those in HFD-fed control mice (data not shown). This result suggests that OB7439 does not normally colonize in mice under physiological conditions.

### 3.5 *Acidipropionibacterium acidipropionici* OB7439 contributed to host metabolic conditions via GPR41

**FIGURE 5:**
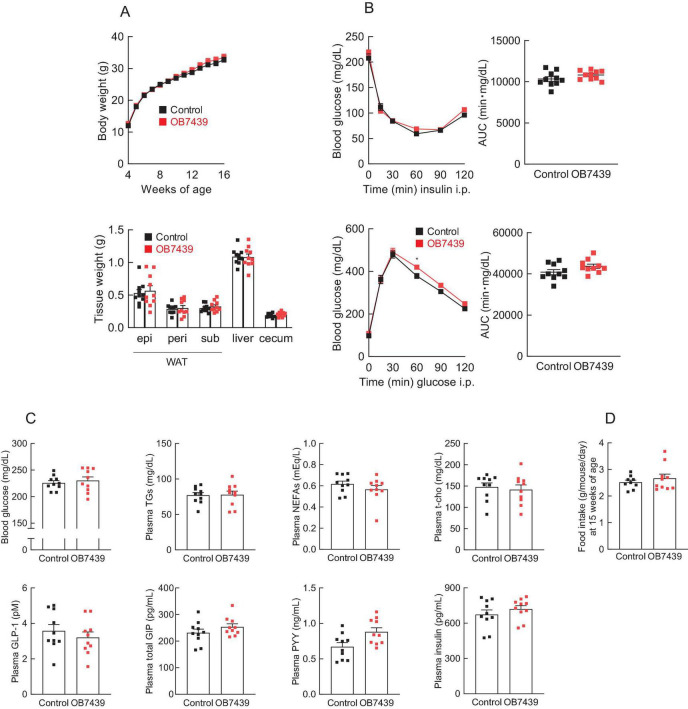
The beneficial effects of *Acidipropionibacterium acidipropionici* OB7439 are attenuated in *Gpr41^–/–^*mice. *Gpr41^–/–^* male mice were fed a high-fat diet (HFD, Control) or *Acidipropionibacterium acidipropionici* OB7439 (1 × 10^7^ cfu/g)-supplemented HFD (OB7439) for 12 weeks, and the beneficial metabolic effects of OB7439 were evaluated as per Figure 3. **(A)** Body and tissue weights. epi, epididymal; peri, perirenal; sub, subcutaneous; WAT, white adipose tissue. **(B)** Insulin tolerance test (ITT) after intraperitoneal (i.p.) injection and area under the curve (AUC) analyses of ITT (*upper*, *n* = 8–10 per group) were performed at 13–14 weeks of age. Glucose tolerance test (GTT) after intraperitoneal (i.p.) injection and AUC analyses of GTT (*lower*, *n* = 8–10 per group) were performed at 13–14 weeks of age. **(C)** Blood glucose, plasma triglycerides (TGs), non-esterified fatty acids (NEFAs), total cholesterol (t-cho), glucagon-like peptide-1 (GLP-1), glucose-dependent insulinotropic polypeptide (GIP), peptide YY (PYY), and insulin levels were measured at the end of the experimental period (*n* = 10 per group). **(D)** The daily food intake at 15 weeks of age (*n* = 9–10 per group). *P < 0.05 (Mann–Whitney U test). Results are presented as the mean ± standard error of the mean (SEM).

SCFAs exhibit various physiological functions related to energy regulation via GPCRs (49). Finally, we investigated whether SCFAs derived from OB7439 improved HFD-induced obesity via GPR41. In *Gpr41^–/–^* mice, the cecal SCFA levels in OB7439-supplemented HFD-fed mice were significantly higher than those in HFD-fed control mice (Supplementary Figure 4). Additionally, suppression of HFD-induced weight and tissue weight gain, insulin resistance, and glucose intolerance by OB7439 supplementation were comparable with that in HFD-fed *Gpr41^–/–^* control mice (Figures 5A,B). Changes in metabolic parameters following OB7439 supplementation were also completely abolished in *Gpr41^–/–^* mice (Figure 5C). However, the food intake was similar between HFD-fed control and OB7439-fed mice (Figure 5D). Therefore, these results indicated that the metabolic effects of OB7439 are related to propionate production and GPR41 signaling.

## 4 Discussion

Viable bacteria, such as probiotics, are involved in regulating host energy homeostasis; however, the underlying molecular mechanisms remain unclear. In this study, we observed that *A. acidipropionici* OB7439–supplemented diet improved HFD-induced obesity via propionate–GPR41 signaling.

Dietary fibers are fermented by gut microbiota and finally converted to SCFAs, such as acetate, propionate, and butyrate. These SCFAs are absorbed via the colonic epithelium. The concentration of acetate, the most abundant SCFA reaches 19–160 μM in peripheral blood, whereas those of propionate and butyrate reach 1–13 μM and 1–12 μM, respectively (50). SCFAs can be incorporated into lipids and glucose, which are the main energy sources for the host (51). We recently reported that SCFAs also function as signaling molecules and regulate energy homeostasis via GPCRs (8). In the present study, OB7439 significantly elevated SCFA levels, especially that of propionate, in bacterial culture supernatants and the cecum. In contrast, the improvement of metabolic dysfunction induced by OB7439 administration was not observed in *Gpr41^–/–^* mice. Hence, our findings suggest that the elevation of propionate level by OB7439 activated GPR41 and enhanced host energy homeostasis.

Dietary fiber is fermented by the gut microbiota to produce SCFAs, including propionate, essential signaling molecules in host energy homeostasis-related physiological functions that act via GPR41 or GPR43 (52, 53). Propionate is a unique activator for both GPR41 and GPR43 (49). Although plasma and cecal acetate were also significantly increased in OB7439-supplemented HFD-fed mice (Figures 1B,C and Supplementary Figure 4), GPR43 is not activated by acetate suggesting that it may contribute to the GPR41-mediated effects (Figure 5). We found that OB7439 suppressed the HFD-induced liver weight gain, hepatic TG accumulation, and changes in the expression of hepatic inflammation- and lipid metabolism-related genes; these effects were abolished in *Gpr41^–/–^* mice. Similarly, a previous study showed that dietary propionate intake suppressed the synthesis of hepatic fatty acids (33). Additionally, 3-(4-hydroxy-3-methoxyphenyl)propionic acid, a metabolite produced by gut microbiota, activated GPR41, contributing to anti-obesity effects and the suppression of hepatic steatosis (34). Although GPR41 expression in the liver is low (27), GPR41 signaling may significantly, albeit indirectly, affect hepatic functions. It is more likely that plasma SCFAs indirectly influence the expression of hepatic inflammation- and lipid metabolic-related genes, such as *Tnf*α and *Fas*, in the liver via other GPR41-expressing tissues. Hence, further studies are warranted to clarify this GPR41-mediated molecular mechanism using tissue-specific GPR41-deficient mice.

Propionibacterium propionate has also been investigated for immunomodulation through the regulation of T-cell subsets.

A commensal Propionibacterium strain mitigated intestinal inflammation through Th17 cell regulation (46).

Probiotic bacteria, such as *Lactobacillus* and *Bifidobacterium*, exert health benefits by altering the gut microbiota (54). Administration of *Lactobacillus acidophilus* together with an HFD, attenuated hyperlipidemia, steatohepatitis, and obesity in a mouse model by improving gut dysbiosis and the production of anti-inflammatory substances (55). In contrast, in this study, we observed that the intake of OB7439 significantly suppressed weight gain in HFD-induced obesity (Figure 2) but did not significantly affect the gut microbiota compositions (Figure 4; Supplementary Figure 3). However, we found that among the gut microbial metabolites, the levels of SCFA in OB7439-fed mice were significantly higher than those in HFD-fed control mice, suggesting that they may contribute to glucose metabolism and anti-obesity effects. In a previous study, functional and mechanistic analyses were performed in gut commensal bacteria to assist in the identification of the key metabolic pathways. The different types of *Acidipropionibacterium* genus and *Bacteroides thetaiotaomicron* are involved in propionate biosynthesis using methylmalonyl-CoA mutase as a key enzyme (56, 57). These results suggested that OB7439 may function as an SCFA producer, but not as a probiotic-like agent, against obesity-induced dysbiosis.

There are some limitations in this study; (1) While we clarified the anti-obese effects through SCFA production in OB7439-fed mice, we did not investigate some detailed metabolism pathways including SCFA production. Additionally, it remains unclear whether SCFA production is a direct effect of OB7439. (2) We found a reduction in the inflammatory genes, but not T cell transcription markers, in the liver of OB7439-fed mice. Because the last 30 years have witnessed a renaissance in the field of immunology and metabolism, comprehensive immunological considerations focusing on metabolic organs will also be important. (3) Moreover, our study primarily relied on murine models, which are valuable for investigating mechanistic pathways but may not fully recapitulate human gut microbiota and host physiology. The extent to which our findings translate to human metabolic regulation remains uncertain, and further validation in human-relevant models is necessary. These gaps raise questions regarding how the intake of OB7439 affects host energy and immune metabolism through SCFA production beyond its effect on anti-obese effects.

In conclusion, we demonstrated that *A. acidipropionici* OB7439 prevented HFD-induced obesity by improving body weight, glucose tolerance, plasma parameters, and hepatic metabolism via propionate production. Importantly, the beneficial effects of *A. acidipropionici* OB7439 were closely associated with self-propionate production and the GPR41–propionate signaling. Our study acts as a reference for the development of potential functional foods supplemented with *A. acidipropionici* OB7439 as an SCFA producer. This will aid in the prevention of metabolic disorders, such as obesity and type 2 diabetes, through a promising therapeutic strategy for improving metabolic disorders.

## Data Availability

The datasets presented in this study can be found in online repositories. The names of the repository/repositories and accession number(s) can be found at: https://www.ddbj.nig.ac.jp/, DRA019204.

## Footnotes

1
http://qiime.org/

2
www.graphpad.com
