# Supplementary material for: Acidipropionibacterium acidipropionici, a propionate-producing bacterium, contributes to GPR41 signaling and metabolic regulation in high-fat diet-induced obesity in mice
Source: Front Nutr. 2025 Apr 3;12:1542196. doi: 10.3389/fnut.2025.1542196 (PMC12003125; doi:10.3389/fnut.2025.1542196)
Supplement: Supplementary file 1 [file Image_1.pdf]

Supplementary Material

Propionate–producing bacteria, *Acidipropionibacterium acidipropionici*, prevents metabolic dysregulation via GPR41 signaling in high-fat diet-induced obese mice

Junki Miyamoto[1*], Yuna Ando[2], Mayu Yamano[3], Akari Nishida[3], Kota Murakami[1], Ikuo Kimura[1,2,3*]

[1]Department of Applied Biological Science, Graduate School of Agriculture, Tokyo University of Agriculture and Technology, Fuchu-shi, Tokyo 183-8509, Japan

[2]Laboratory of Molecular Neurobiology, Graduate School of Biostudies, Kyoto University, Kyoto 606-8501, Japan

[3]Department of Molecular Endocrinology, Graduate School of Pharmaceutical Sciences, Kyoto University, Kyoto 606-8501, Japan

*** Correspondence:**

Junki Miyamoto
m-junki@go.tuat.ac.jp

Ikuo Kimura
kimura.ikuo.7x@kyoto-u.ac.jp

Keywords: ***Acidipropionibacterium acidipropionici*, short-chain fatty acids, propionate, obesity, GPR41.**

1 Supplementary Figures and Tables

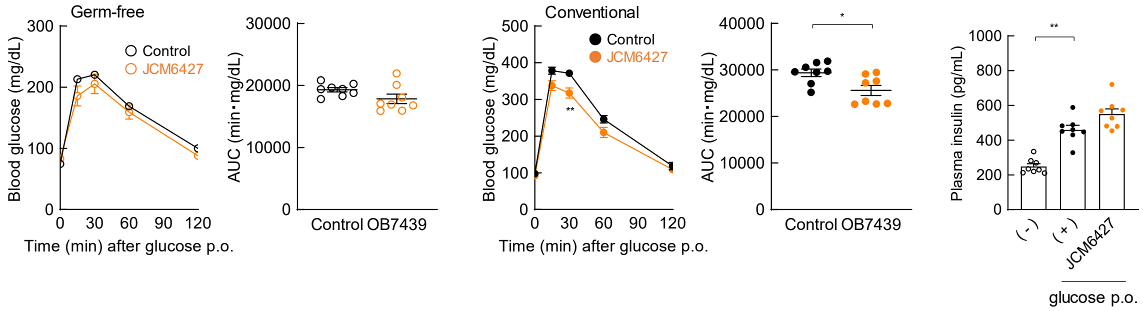

Supplementary Figure S1. Regulation of glucose homeostasis by *Acidipropionibacterium acidipropionici* JCM6427.

(**A**) Blood glucose levels in germ free (GF)-ICR (n = 8 per group) and (**B**) conventional C57BL/6J (n = 8 per group) mice according to oral glucose tolerance test (GTT) and area under the curve (AUC) analyses of GTT performed after 16-h fasting, as shown in Fig. 2. $^{**}P < 0.01$ and $^{*}P < 0.05$, compared to control (Mann–Whitney test). (**C**) Plasma insulin levels were measured 30 min after oral glucose administration (p.o) in C57BL/6J mice (n = 8 per group). (-) indicates only fasting, and (+) indicates only glucose p.o.. *Acidipropionibacterium acidipropionici* JCM6427 (JCM6427; 1×10^{10} cfu/mouse). $^{**}P < 0.01$ (Dunn's post-hoc test). Results are presented as the mean ± standard error of the mean (SE).

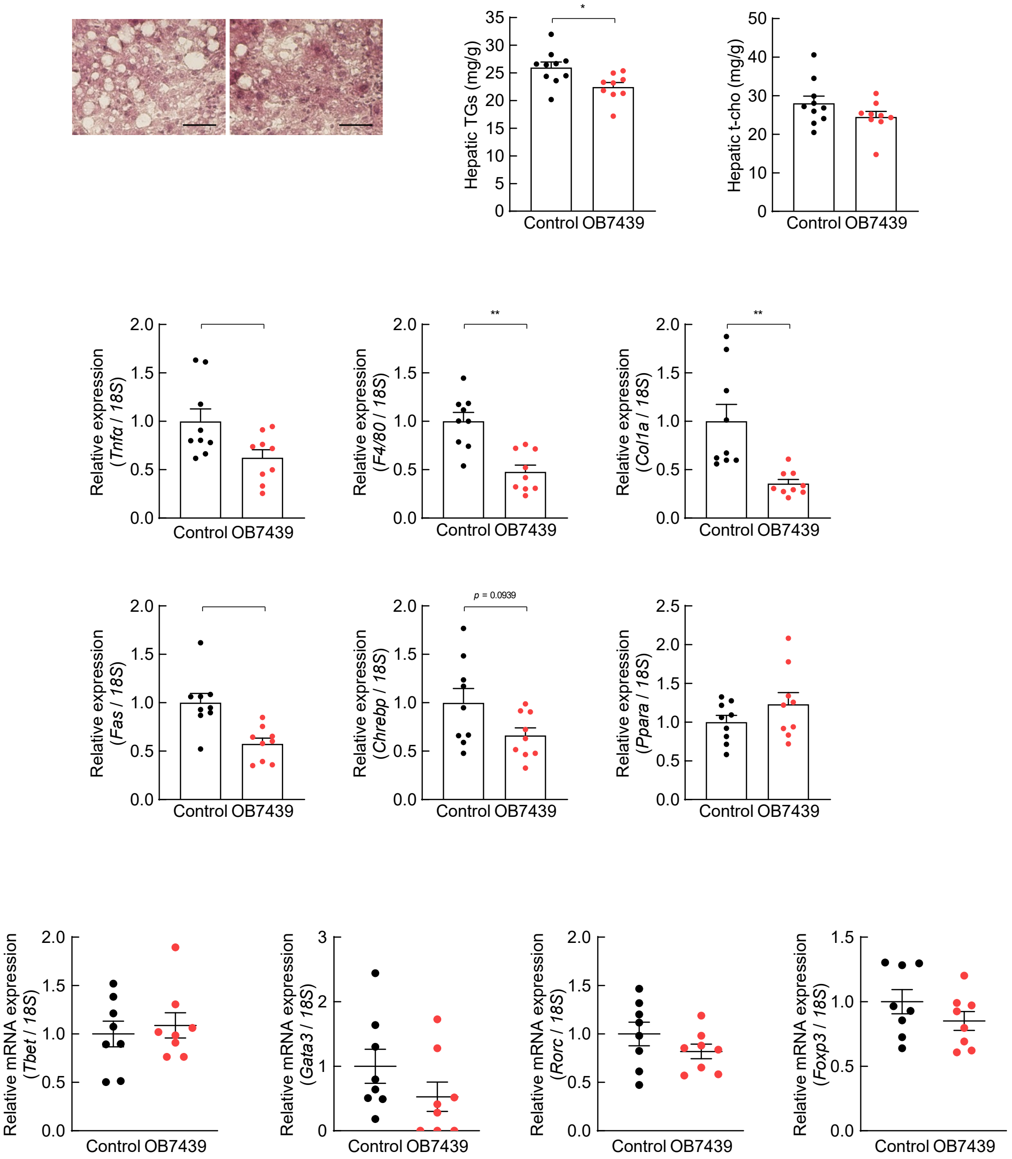

Supplementary Figure 2. *Acidipropionibacterium acidipropionici* OB7439 improves host metabolic condition in the liver.

C57BL/6 J male mice were fed a high-fat diet (HFD, Control) or an *Acidipropionibacterium acidipropionici* OB7439 (1 × 10^7 cfu/g)-supplemented HFD (OB7439) for 12 weeks. (**A**) Hepatic histology using hematoxylin and eosin (H&E) staining; Scale bar, 200 µm. (**B**) Hepatic triglycerides (TGs) and total cholesterol (t-cho) content in the liver (n = 9–10 per group). (**C, D**) The mRNA expression levels of hepatic genes related to inflammation and energy metabolism (**C**) and T cell

transcription markers (**D**) were measured using RT-qPCR (n = 8–10 per group). $^{**}P < 0.01$ and $^{*}P < 0.05$ (Mann–Whitney U-test). Results are presented as the mean ± standard error of the mean (SEM).

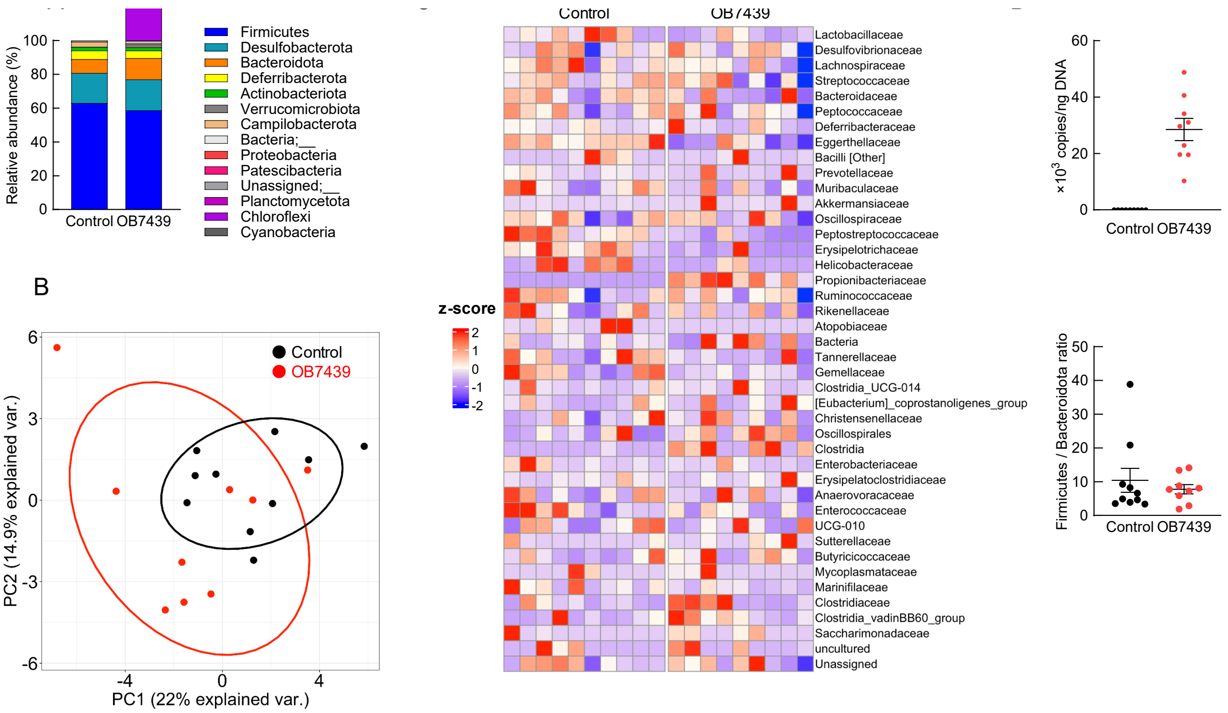

Supplementary Figure 3. ***Acidipropionibacterium acidipropionici*** **OB7439 changes gut microbial composition in the feces of obese mice.**

(**A**) Relative abundance of gut microbiota at the phylum level, (**B**) principal coordinate analysis, (**C**) abundance of gut microbiota at the family level, (D) levels of *A. acidipropionici*, and (E) Firmicutes to Bacteroidota ratio (n = 9–10). Data are presented as means ± standard error of the mean (SEM). Control, mice fed high-fat diet (HFD); OB7439, mice fed HFD supplemented with *Acidipropionibacterium acidipropionici* OB7439 (1 × 10^7 cfu/g).

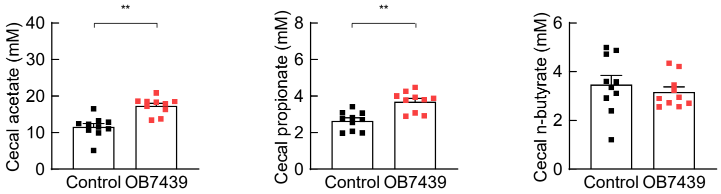

Supplementary Figure 4. Short-chain fatty acid (SCFA) production in *Gpr41*$^{-/-}$ mice.

SCFA levels in the cecum of high fat diet (HFD)-fed mice (n = 10 per group); OB7439, HFD diet with OB7439 supplementation (1×10^7 cfu/g); Control, HFD diet without OB7439 supplementation. All SCFA levels were measured using GC-MS. **$P < 0.01$, *$P < 0.05$ in Mann–Whitney test. Results are presented as the mean ± standard error of the mean (SEM).
